# Deep-Learning-Based Wi-Fi Indoor Positioning System Using Continuous CSI of Trajectories

**DOI:** 10.3390/s21175776

**Published:** 2021-08-27

**Authors:** Zhongfeng Zhang, Minjae Lee, Seungwon Choi

**Affiliations:** Department of Electronic Engineering, Hanyang University, Seoul 04763, Korea; zhongfeng.zhang@dsplab.hanyang.ac.kr (Z.Z.); minjae.lee@dsplab.hanyang.ac.kr (M.L.)

**Keywords:** Wi-Fi IPS, trajectory CSI, 1DCNN-LSTM, GAN

## Abstract

In a Wi-Fi indoor positioning system (IPS), the performance of the IPS depends on the channel state information (CSI), which is often limited due to the multipath fading effect, especially in indoor environments involving multiple non-line-of-sight propagation paths. In this paper, we propose a novel IPS utilizing trajectory CSI observed from predetermined trajectories instead of the CSI collected at each stationary location; thus, the proposed method enables all the CSI along each route to be continuously encountered in the observation. Further, by using a generative adversarial network (GAN), which helps enlarge the training dataset, the cost of trajectory CSI collection can be significantly reduced. To fully exploit the trajectory CSI’s spatial and temporal information, the proposed IPS employs a deep learning network of a one-dimensional convolutional neural network–long short-term memory (1DCNN-LSTM). The proposed IPS was hardware-implemented, where digital signal processors and a universal software radio peripheral were used as a modem and radio frequency transceiver, respectively, for both access point and mobile device of Wi-Fi. We verified that the proposed IPS based on the trajectory CSI far outperforms the state-of-the-art IPS based on the CSI collected from stationary locations through extensive experimental tests and computer simulations.

## 1. Introduction

Owing to an increase in the number of portable devices, such as mobile phones and tablets, over the past few years [1,2], an increasing number of indoor location-based services [3], including navigation, location-based social networking, and motion tracking have attracted more and more attention over time. Unlike the outdoor environment, where the global positioning system (GPS) can provide accurate localization using line-of-sight signals, the indoor positioning system based on GPS significantly degrades because of signal attenuation through the building’s walls [4]. Compared to GPS signals, Wi-Fi signals are more stable in indoor environments because of their wide deployment and easy access; thus, the utilization of Wi-Fi signals to achieve accurate indoor localization has recently gained significant interest [5].

As a state-of-the-art technology, Wi-Fi fingerprinting indoor localization systems (IPS) have been extensively researched for both localization [6,7] and activity recognition [8,9] applications. As the radio frequency (RF) characteristics of Wi-Fi signals at each location are unique due to their different propagation paths, the RF characteristics can be considered unique fingerprints. With all the fingerprints of locations collected and stored in the database beforehand, an accurate localization can be achieved by comparing the received Wi-Fi signal with the data in the database.

A Wi-Fi fingerprinting IPS can utilize either the received system strength indicator (RSSI) or channel state information (CSI) as fingerprints for each location. For Wi-Fi-based IPSs using RSSI, ref. [10] demonstrated that an RSSI heat map can be used to estimate the target location by applying the overlap technique to RSSI heat maps of access points (APs). The heat maps generated for both line-of-sight and non-line-of-sight (NLOS) path loss models for each AP for the indoor environment. However, how to accurately select the proper path loss models for a given complex indoor environment to construct the accurate RSSI heat maps is a challenge since the localization accuracy completely relies on the accuracy of the RSSI heat map, which depends on the accuracy of the signal propagation path loss model selected for each location on the map. Compared to RSSI-based IPSs, CSI-based IPSs are preferred for the following reasons [11]. First, the RSSI varies constantly with time due to fading and multipath effects in indoor environments [12], making the system unreliable. The unreliability can cause severe localization errors, even when the target device remains stationary. Second, the information extracted from the RSSI is extremely limited because the RSSI is simply the strength indicator of the received signal, which is highly subject to environmental interference. Due to these interferences, the RSSI may appear similar even for completely different locations due to the complex indoor signal environment. Third, to achieve a more accurate localization, RSSI-based IPS requires numerous APs [13], which may not be available in many practical circumstances, such as ordinary houses, small stores, and offices. In contrast to RSSI, CSI is more stable and provides richer location-related information from multiple subcarriers employed in orthogonal frequency-division multiplexing (OFDM) signals. By exploiting the fine-grained multipath information from OFDM signals in the physical layer (PHY), the CSI-based IPS can achieve accurate localization with only a single AP.

Despite all the merits and preferences in localization applications, the Wi-Fi fingerprinting IPS still requires significant improvements to be practical. To resolve these problems in CSI-based IPS, we consider the following three key aspects in this paper.

First, we investigate data collection procedure to provide specific information representing the unique characteristics of each location of interest—the collected data should be stable and trustworthy, such that the system utilizing the data can be sufficiently robust. The conventional method of collecting data for Wi-Fi fingerprinting IPS measures the CSI from arbitrarily stationary locations predetermined in advance [14,15,16,17]. The operation of conventional CSI-based IPS assumes that the CSI measured at a test point (TP) should be more highly correlated to that of the most nearby reference point (RP) than to that of all the other RPs. However, we found that the correlation is unreliable through extensive experiments, mainly due to the NLOS indoor environment. The correlation-based conventional methods utilizing the CSI collected from preset stationary locations result in many ambiguities in localization accuracy. Consequently, there is a need for a novel method of collecting CSI that can present distinctive features for each location.

Second, we designed a system that learns the underlying information from the inputs as effectively and efficiently as possible. Many IPS-related papers [18,19,20] that utilize deep learning solutions claim to have achieved excellent performance with an error of less than 1 m; however, these results can be obtained only when the test data and training data are collected from the same location. In other words, if the test data were measured from a random position, i.e., unknown to the deep learning neural network during the training period, the performance of the IPS would seriously degrade and fail to meet the accuracy claimed in their works; therefore, the application of such IPSs in practice is highly limited because the target devices do not always remain at predetermined locations. Moreover, the complexity of the system should be considered, which is important for feasible implementation. In [20,21], despite the excellent performances claimed in their IPSs, their complexity has not been addressed, which lowers their practical value. Based on the discussions mentioned above, there is a need to design an IPS that requires a reasonable complexity without compromising its excellent performance.

Third, we explore methods to efficiently acquire more training data at a reasonable cost. With more training data, a more accurate neural network system that provides better mapping between the input and output without overfitting can be obtained; however, acquiring more data is time-consuming and labor-intensive [22]. Many studies [23,24,25] have attempted to reduce the data collection cost by either reducing the number of RPs or recovering the damaged fingerprints during data collection, which inherently affects the IPS performance. Consequently, to reduce the cost required for data collection and obtain more data that can ensure the IPS’s excellent accuracy, there is a need to find a novel method to efficiently enlarge the dataset without actually measuring the data.

In this paper, we address the three key aspects to optimize the IPS by proposing a novel method of measuring CSI along trajectories instead of collecting the CSI from predetermined stationary locations. Compared to the CSI collected from a stationary location, the continuous CSI collected from a trajectory provides both the CSI from the current location and the CSI from previous locations. To exploit both the spatial and temporal information from the trajectory CSI, we adopted a one-dimensional convolutional neural network–long-short term memory (1DCNN-LSTM) neural architecture to enhance the accuracy of the proposed IPS. We also employed a generative adversarial network (GAN) to resolve the challenges of obtaining more training data. The excellent performance of the proposed IPS is observed through extensive experiments using a testbed implemented with multicore digital signal processors (DSPs) and a graphics processing unit (GPU) for the Wi-Fi emulator and neural network system, respectively.

The main contributions of this paper can be summarized as follows.

- Proposal of adopting continuous CSI captured along each trajectory instead of stationary RPs in a given indoor environment as a novel feature of creating a fingerprinting map for IPS.
- Hardware implementation of the entire IPS using DSPs as an emulator of Wi-Fi APs and mobile devices (MDs), and a GPU as a trainer for the 1DCNN-LSTM deep learning architecture.
- Performance analysis of a 1DCNN-LSTM deep learning architecture as a function of the filter size, number of layers, batch size, etc.
- Application of the GAN to enlarge the dataset such that a large amount of synthetic trajectory CSI can be generated to be added as input data of the 1DCNN-LSTM deep learning architecture without actually collecting the trajectory CSI, which consequently enhances the performance of the proposed IPS with limited dataset size.

The remainder of this paper is organized as follows. In Section 2, an IPS based on CSI is introduced. In Section 3, the proposed data collection method is discussed and compared with the traditional data collection method. In Section 4, the implementation of the IPS is presented. In Section 5, the detailed deep learning solution, including the proposed deep neural network, is explained. In Section 6, the experimental results are presented and analyzed. In Section 7, the conclusions of the paper are summarized.

## 2. System Model

### 2.1. Channel Analysis Using CSI

CSI contains fine-grained information of the wireless channel, especially for OFDM-based systems, because it can be separately obtained for each subcarrier, where RSSI simply provides coarse-grained information for the entire frequency band [11]. Further, due to the RF front-end impairment between the transmitter and each receiver, which differs per location, the CSI is preferred to construct a unique fingerprint map for each indoor location.

Let $\vec{T}$ and $\vec{R}$ denote the transmit (Tx) and receive (Rx) signal vectors generated from the RF transceiver. In this paper, we utilize a universal software radio peripheral (USRP), which is a reconfigurable RF transceiver including a field-programmable gate array, to generate Wi-Fi signals in the 2.4 GHz band. Thus, the Rx signal can be written as:

(1) $$\vec{R}=\vec{H}\cdot \vec{T}+\vec{N},$$

where $\vec{N}\;$ denotes the additive white Gaussian noise vector with $\vec{H}$ being the channel, which can be acquired from the CSI.

The ith subcarrier channel $H_{i}$ is a complex-valued quantity that can be written as:

(2) $$H_{i}=\left|H_{i}\right|e^{j∠H_{i}},$$

where |$H_{i}$| and $∠H_{i}$ are the amplitude and phase of the channel for the ith subcarrier, respectively.

Only the amplitude of the channel, $H_{i}$, is considered, ignoring the phase information owing to random jitters and noise caused by the imperfect hardware in the RF transceiver [16].

### 2.2. High-Level Design of IPS

**Figure 1:**
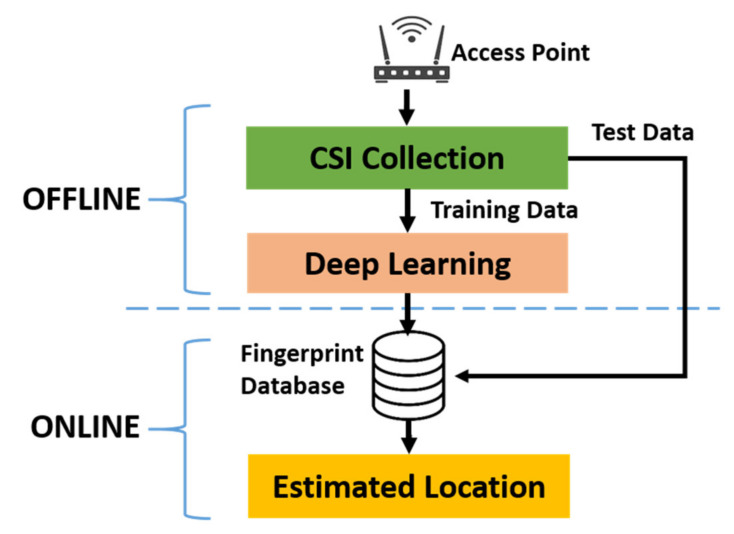
The indoor positioning system structure.

The IPS implementation procedure can be divided into two phases, offline and online, as shown in Figure 1.

During the offline phase, the CSI at each predefined RP was collected and used as training data for the deep learning neural network. Note that, depending on various performance requirements, a deep learning network structure can be customized in many ways [20]. After training the deep learning neural network, the resultant network structure and the weights of the network neurons are stored in the fingerprint database to be used during the online phase.

During the online phase, the CSI at each predefined TP, which is generally different from the RPs, is collected and used as test data to evaluate the performance of the entire IPS designed during the offline phase, as described above.

In this paper, only a single AP with a single Tx antenna was considered, along with several predefined RPs in an area of interest. Each RP $p_{i}$, at its physical location $p_{i}(x_{i},\;y_{i})$, acquires the corresponding CSI received from the AP. For each RP $p_{i}$, we collected N CSI amplitude measurements, each of which comprised observations at W subcarriers. Consequently, the resultant N×W measurement matrix can be written as:

(3) $$A_{p_{i}}=\left(\begin{array}{ccc} a_{i}^{11}\;a_{i}^{12}\;a_{i}^{21}\;a_{i}^{22} & \cdots & a_{i}^{1W}\;a_{i}^{2W}\\ \vdots & \ddots & \vdots \\ a_{i}^{N1}\;a_{i}^{N2} & \cdots & a_{i}^{NW} \end{array}\right)$$

where $a_{i}^{nw}$ is the CSI amplitude value for the subcarrier *w* in measurement n at each RP $p_{i}$.

## 3. Data Preparation

### 3.1. Conventional Data Collection Method

**Figure 2:**
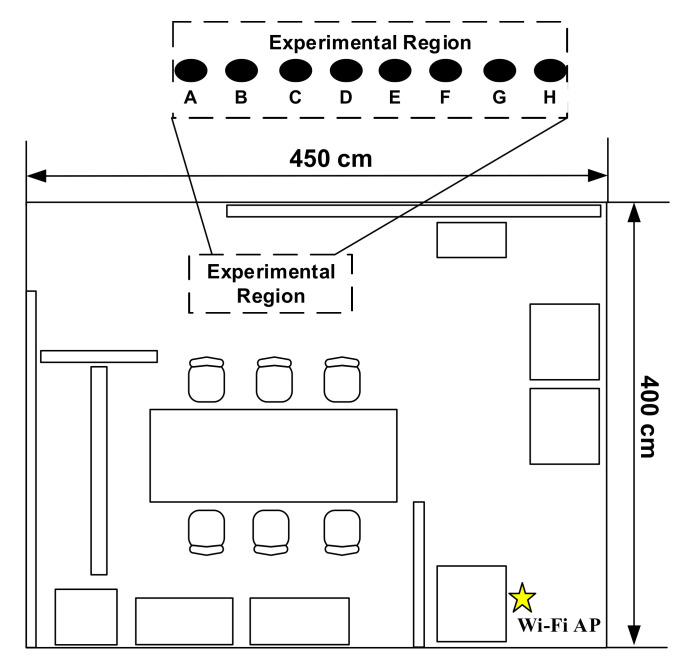
Indoor laboratory with eight RPs in a selected experimental region and a yellow star representing Wi-Fi AP.

**Figure 3:**
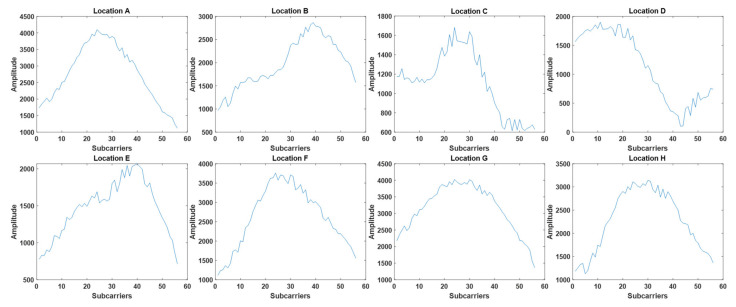
CSI received at different locations.

The conventional method of collecting channel data is based on the CSI measured from each stationary location [16,17,18,19,20], which is predetermined in a given fingerprint map. To observe the characteristics of the channel data collected from adjacent locations using the conventional data collection method, we first experimented in our laboratory (7.5 m × 3 m), as shown in Figure 2. The NLOS effect on signal propagation is incurred by the walls, cabinets, tables, and other partitions in the experimental environment. We selected an experimental region with eight RPs, as shown in Figure 3, where each RP was uniformly separated with a spacing of 5 cm. The eight RPs are represented as black points with indices from A to H. The AP, which is depicted as a yellow star, transmits a Wi-Fi signal, and the Rx antenna at each of the eight RPs collects the channel data based on the CSI included in the Wi-Fi signal. The channel data comprise 56 complex values carried by 56 subcarriers in each OFDM symbol of the Wi-Fi signal.

Figure 3 illustrates the instantaneous CSI measurements obtained at the eight RPs, A~H, in the experimental region shown in Figure 2. Although the CSI measurements of adjacent RPs are expected to appear quite similar to each other, the similarity between adjacent CSI compared to that between non-adjacent CSI is not so conspicuous that the CSI correlation obtained from adjacent RPs is not generally higher than that obtained from non-adjacent RPs.

**Figure 4:**
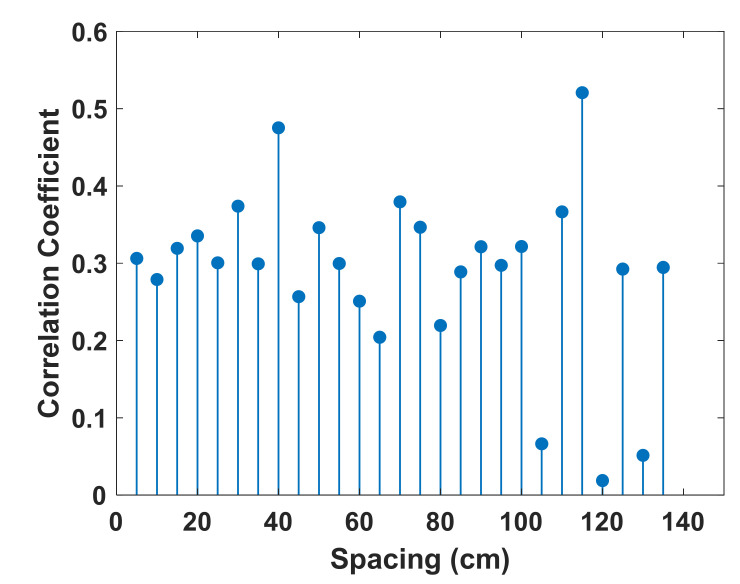
Correlation coefficient of CSI between two RPs as a function of spacing.

For an extensive comparison, Figure 4 shows the average CSI correlation coefficient as a function of the RP spacing, where the RP spacing ranges from 5 to 135 cm. The average correlation coefficient with spacing $s_{i}$ is defined as:

(4) $$\rho \left(s_{i}\right)=\frac{\sum_{k=1}^{N}\rho_{k}\left(s_{i}\right)}{N},$$

where N is the number of CSI measurements between every pair of RPs with spacing $s_{i}$, and $\rho_{k}\left(s_{i}\right)$ is the Pearson coefficient [26] corresponding to every pair of RPs with spacing $s_{i}$. From Figure 4, when the spacing is less than 100 cm, the CSI correlation is not proportional to the distance between the RPs. When the spacing is larger than 100 cm, the CSI correlation generally decreases; however, there is still a relatively high correlation when the spacing is equal to 115 cm. The result indicates that a smaller spacing does not necessarily result in a high CSI correlation compared to a larger spacing. The CSI correlation is almost independent of the spacing, which is mainly owing to the NLOS effects. From the observations obtained from the indoor experimental tests shown in Figure 2, it is concluded that CSI measurements from a set of predetermined RPs cannot be used as reliable channel data for designing an IPS with high localization accuracy. Although it might still be feasible to achieve an acceptable accuracy by collecting CSI measurements with extremely small spacing, which could be less than 5 cm, the data collection is undoubtedly inefficient and time-consuming.

### 3.2. Proposed Data Collection Method

In this subsection, we propose a novel paradigm of collecting the channel data, which makes it possible to collect channel data from the trajectory in between each pair of RPs. As the conventional method of collecting the channel data is focused on stationary RPs only, it can only collect channel data from the predetermined location points, which causes the channel data in between RPs to be ignored. Consequently, the proposed technique allows channel data captured continuously between each pair of adjacent RPs, instead of separately collecting the channel data at each RP.

**Figure 5:**
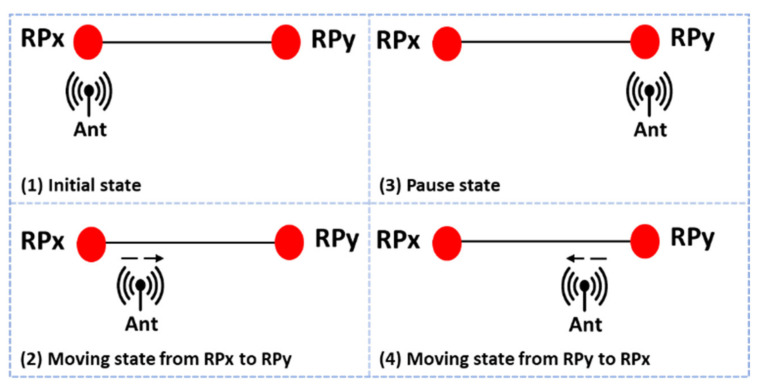
Proposed data collection process to capture the channel data along a trajectory between two RPs ($RP_{x}$ and $RP_{y}$).

The proposed data collection process comprises four steps to capture the channel data along with each trajectory between two RPs ($RP_{x}$ and $RP_{y}$) as shown in Figure 5. First, an initial state in which the Rx antenna remains at $RP_{x}$ for a short time is observed. Second, a moving state in which the Rx antenna moves along the trajectory from $RP_{x}$ to $RP_{y}$ is observed. During this state, the Rx antenna continuously captures the Wi-Fi signal transmitted from the AP. Third, a motionless state in which the Rx antenna remains still for a short time after arriving at $RP_{y}$ before returning to $RP_{x}$ is observed. Finally, the Rx antenna is to move back from $RP_{y}$ to $RP_{x}$ with the Wi-Fi signal being continuously captured. As will be discussed later, the moving speed of the Rx antenna between two RPs is not a critical issue in the proposed data collection process. The channel data collection described above for capturing the trajectory CSI can be performed as many times as desired for each trajectory. The multiple CSI observations per trajectory will be used when training the deep neural network in Section 5.

**Figure 6:**
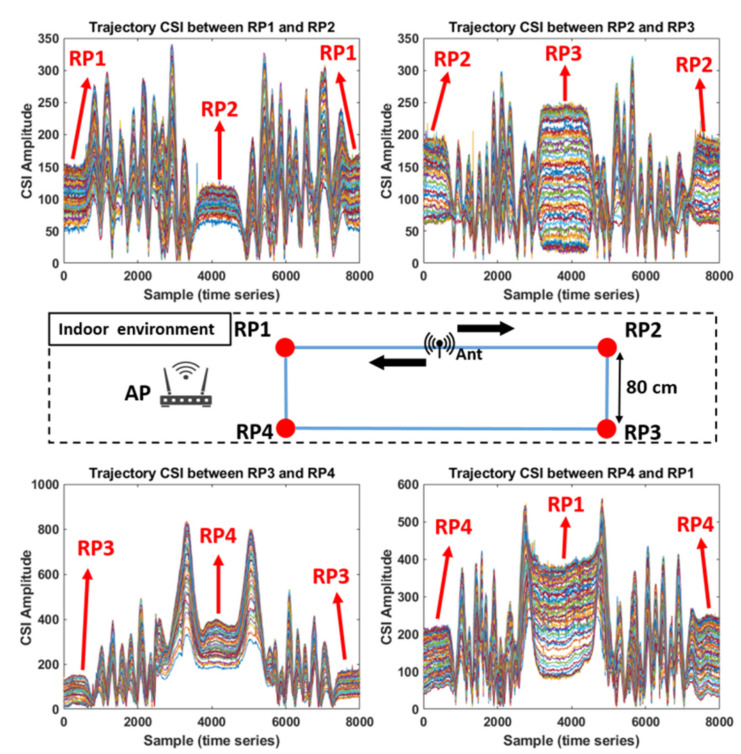
Measurements of the trajectory CSI collected from four trajectories in the indoor environment shown in Figure 2.

Figure 6 illustrates the trajectory CSI collected from four trajectories involved with four RPs in an indoor environment. Each of the four RPs represented in red points is spaced at 80 cm, and the four trajectory CSIs are collected manually through the process shown in Figure 5 from the trajectories between $RP_{1}$ and $RP_{2}$, $RP_{2}$ and $RP_{3}$, $RP_{3}$ and $RP_{4}$, and $RP_{4}$ and $RP_{1}$. Note the reason for choosing 80 cm as the spacing between RPs is that the 80 cm spacing is approximately an adult’s walking step length; therefore, using 80 cm as the spacing is reasonable to simulate the real situation of people moving in the indoor environment. Each of the different colored curves of the trajectory CSI shown in Figure 6 corresponds to each of the 56 subcarriers in a given OFDM symbol. From the figure, a trajectory CSI comprises two observations, one being the measurement obtained from $RP_{x}$ to $RP_{y}$ and the other being the measurement obtained in the other direction, i.e., from $RP_{y}$ back to $RP_{x}$. As the two observations are obtained from the same trajectory in the opposite direction, each trajectory CSI results in a quasi-symmetrical shape. For example, the trajectory CSI measured from $RP_{1}$ to $RP_{2}$ is approximately symmetrical to that measured from $RP_{2}$ to $RP_{1}$ as the CSI collection occurs in two opposite directions in the same trajectory. It can also be observed that quasi-flat curves appear in between the two observations for each trajectory CSI corresponding to the motionless state described in the first and third steps in Figure 5, where the Rx antenna stays still for a short time. By using the quasi-flat curves, the beginning and ending times of each observation in the trajectory CSI measurements that include multiple observations for a given trajectory could be found. Note that the trajectory CSI should be collected as many times as possible to be used as training inputs for the deep learning neural network discussed in Section 5.

**Figure 7:**
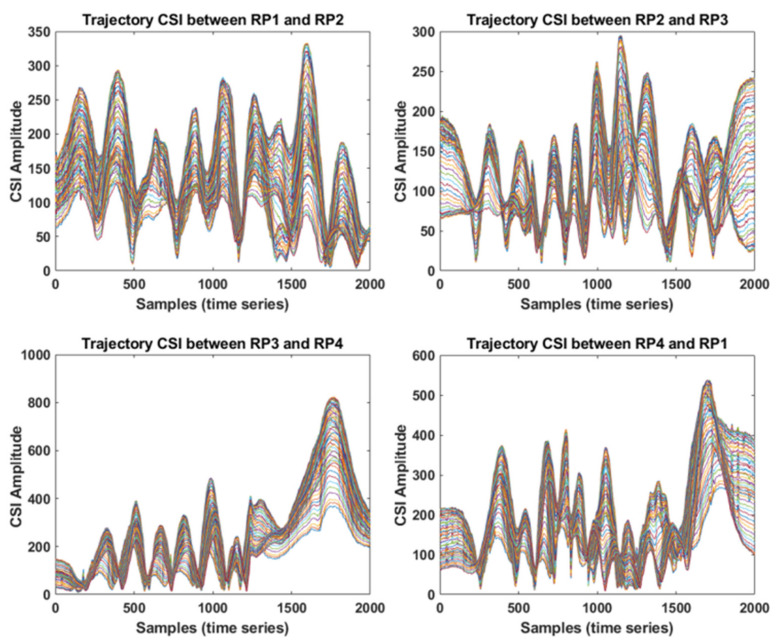
Trajectory CSI observations corresponding to the four trajectories shown in Figure 6.

Figure 7 illustrates a single-trajectory CSI observation extracted from the corresponding CSI measurement, as shown in Figure 6. As the amplitude shapes of each trajectory CSI for the four trajectories are all different from one another, the difference among trajectory CSIs can be used as a key feature for constructing a fingerprinting-based IPS. Note that, from our experiments, it was found that human movements do not seriously affect the amplitude shapes of trajectory CSI unless the Wi-Fi signal at either Tx AP or Rx MD is intentionally blocked. In addition, the movements of small objects such as cups, laptops, notebooks etc., do not seriously affect the amplitude shapes of trajectory CSI either. However, after larger objects such as tables, cabinets, desks etc., near the Tx AP or Rx MD have been moved, the measurements of trajectory CSI can be considerably changed, which could result in completely different amplitude shapes of CSI of each trajectory. In this case, the trajectory CSI would have to be measured again. Although an inconsistent measurement speed while moving between RPs results in a different length of received samples, it can easily be normalized by applying the resampling technique [20]; thus, as shown in Figure 7, the observation length of each trajectory CSI was set to 2000 by adopting the resampling procedure.

From the above discussions, it can be concluded that the proposed channel data based on trajectory CSI corresponds to an aggregation of CSI measurements collected from every stationary point between two RPs. Thus, with the proposed data collection method, it is possible to exhaustively collect all the channel data existing between two RPs using a single CSI measurement.

### 3.3. Predetermined Routes in the Experimental Environment

**Figure 8:**
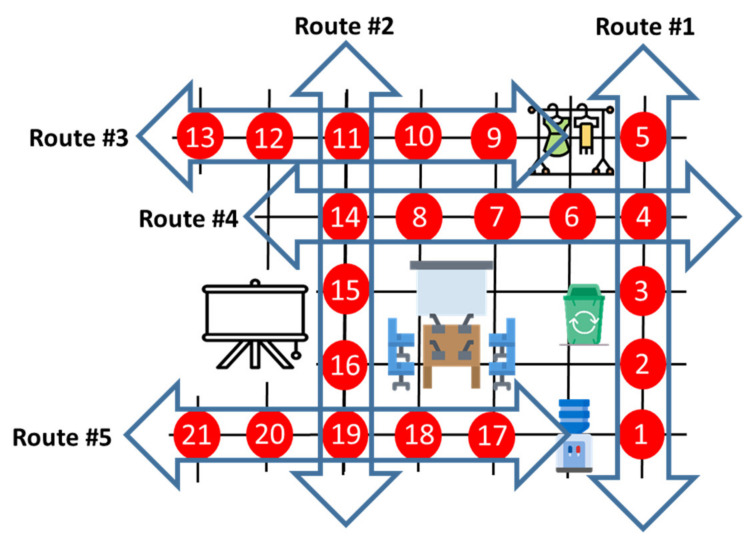
Detailed illustration of the experimental environment comprising five predetermined routes based on the 21 RPs.

To evaluate the performance of the proposed IPS based on the trajectory CSI, we set up five routes, each comprising five adjacent RPs with a uniform spacing of 80 cm in an indoor environment, as shown in Figure 8. Each route includes several trajectories; for example, route #2 comprises five RPs, which provide a series of four consecutive CSI trajectories. With the consecutive CSI trajectories in each route, the CSI observation can be extended from the unit of a single trajectory to that of a route comprising multiple trajectories. Each of the five routes was set up so that all the paths that people normally choose to walk along were covered. Although only five routes were considered in our experiment, the number of routes can be arbitrarily determined based on the actual situation of the experimental environments.

**Figure 9:**
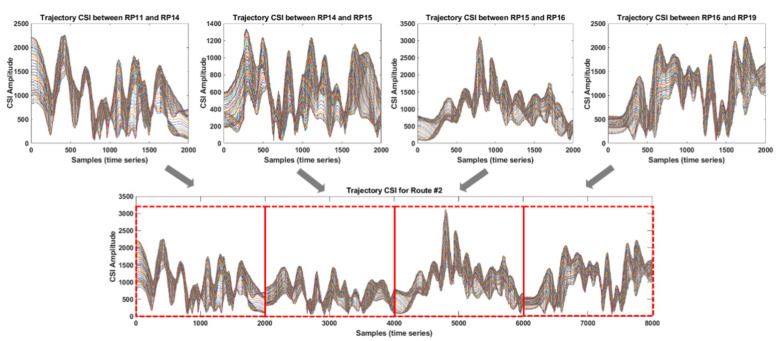
CSI observation extended by concatenating a set of trajectory CSI in a given route.

In this paper, we propose utilizing all trajectory CSI involved in each route; this means that the proposed IPS is designed based on a combination of all trajectory CSI in each route instead of a single CSI trajectory. Thus, the CSI observed along each of the five routes shown in Figure 8 was generated by concatenating all the trajectory CSI involved in each route. Figure 9 illustrates the CSI observation obtained by concatenating the four consecutive CSI trajectories of route #2. Consequently, the resultant CSI comprising multiple consecutive CSI trajectories obtained from each route can be viewed as single-trajectory CSI collected directly along the entire route.

This method of concatenating the set of trajectory CSI provides the IPS with excellent scalability without having to collect the CSI for each route entirely. As the number and complexity of routes in a given experimental environment increase, the abovementioned scalability benefited from the proposed method, which provides more superiority in efficiently generating the CSI for all the desired routes. Further, because the CSI of all the previous locations can be exploited as historical information concerning the current location, the scalability provided by the proposed method greatly contributes to reducing the localization error of the IPS. As will be discussed in subsequent sections, the results of our experiments and computer simulations show that, instead of training the neural network with a dataset composed of the CSI of a single trajectory, training it with a dataset comprising concatenated trajectory CSI significantly enhances the performance of the IPS.

## 4. Hardware Implementation of the Proposed IPS

This section introduces a hardware implementation of the proposed IPS comprising a Wi-Fi encoder emulating the AP and Wi-Fi decoder emulating the MD, which supports the IEEE 802.11ac protocol [27]. A multicore DSP and USRP were used as the modem and RF transceiver, respectively, in both the Wi-Fi encoder and the Wi-Fi decoder.

**Figure 10:**
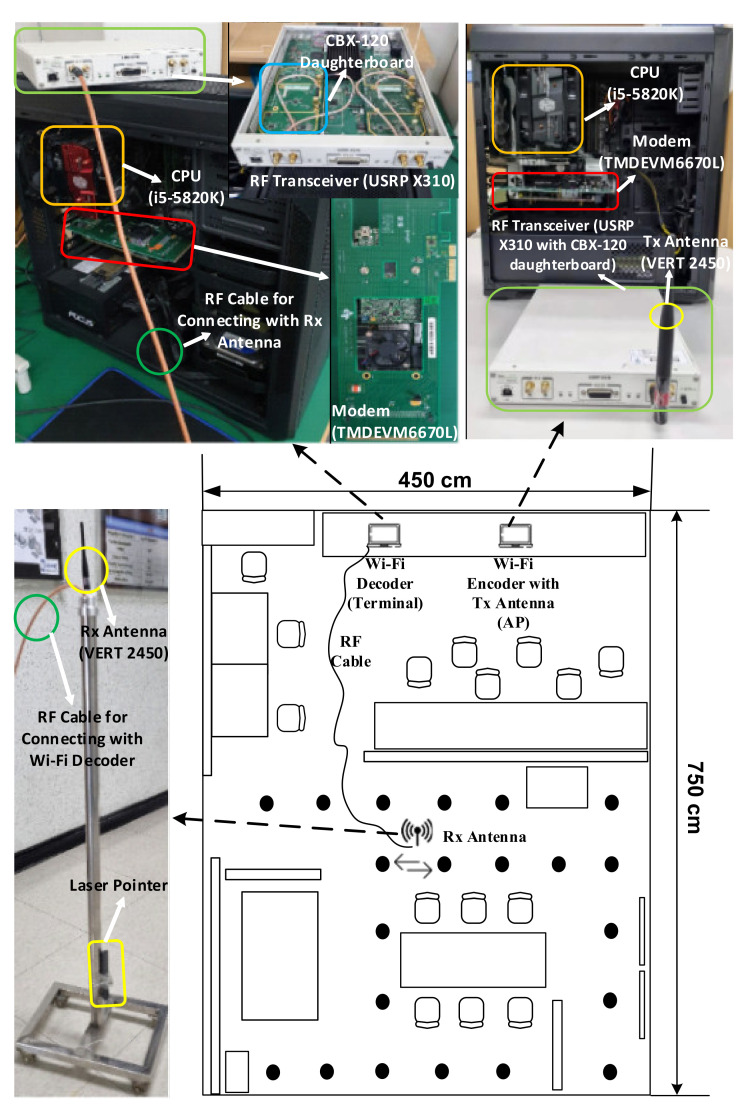
Hardware implementation of the proposed IPS for an indoor environment.

The overall IPS implementation, comprising an encoding AP and decoding MD in an indoor environment, is illustrated in Figure 10. The implemented AP transmits a Wi-Fi downlink (DL) signal to the MD to receive it. The Tx AP comprises the following four parts: an i5-5820k central processing unit to control the Tx AP; a TMDEVM6670L [28] modem to encode the Tx Wi-Fi data; a USRP Ettus X310 RF transceiver with CBX-120 daughterboard [29] for digital-to-analog conversion and frequency up-conversion; and an omnidirectional antenna VERT2450 to radiate the Wi-Fi DL signal, which is denoted in orange, red, green, and yellow colors, respectively. The Rx MD comprises four parts, of which the functionalities are the same as for the Tx AP, except that the MD receives the Wi-Fi signal and the AP transmits the Wi-Fi signal. Note that the Rx antenna connected to the MD via an RF cable was installed on a movable stand such that the CSI could be collected at any desired position; therefore, the movable MD antenna can collect the CSI for any desired trajectory between RPs, 21 of which are denoted as black points in Figure 10. To track the trajectory as accurately as possible while collecting the CSI, a laser pointer was strapped at the bottom of the movable Rx antenna stand.

## 5. Deep Learning Solutions

### 5.1. One-Dimensional Convolutional Neural Network

We adopted a one-dimensional convolutional neural network (1DCNN) to extract useful spatial features from the trajectory CSI needed to design the target IPS. Unlike a two-dimensional convolutional neural network that processes 2D data such as images, the 1DCNN is used for processing 1D data such as the trajectory CSI.

**Figure 11:**
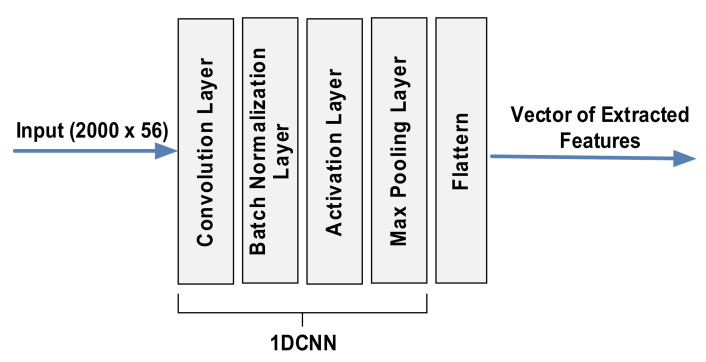
Detailed structure of 1DCNN model.

Figure 11 illustrates a 1DCNN model adopted in the proposed IPS, which includes multiple 1DCNNs, each of which comprises a convolutional layer, a batch normalization layer, an activation layer, and a max-pooling layer. In each 1DCNN, the convolutional layer comprises optimized filters of size 1 × 56 for filtering the input data of size 2000 (the number of samples per trajectory) × 56 (the number of subcarriers per OFDM symbol). The batch normalization layer is applied between the convolutional layer and the activation layer to reduce the sensitivity in setting the network’s initial parameters and fast training [30]. In the activation layer, a nonlinear transfer function, the rectified linear unit, was applied to provide nonlinearity, which is important for the neural network to produce a nonlinear decision boundary via nonlinear combinations of weights and inputs. The output is then down-sampled by passing it through the max-pooling layer to make the resultant features appropriately compact. After being downsampled, the resultant vector is flattened as a vector representing the spatial features extracted from the input trajectory CSI.

### 5.2. 1DCNN-LSTM Architecture

**Figure 12:**
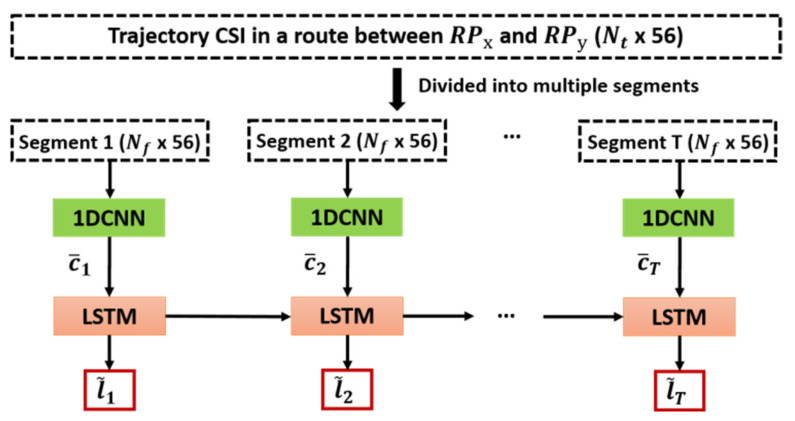
1DCNN-LSTM model adopted for the proposed IPS.

While the 1DCNN is exploited to extract the spatial features of the trajectory CSI, the proposed IPS employs the long and short-term memory (LSTM) [31] to extract the temporal characteristics of the trajectory CSI, as shown in Figure 12. The LSTM is a variation of recurrent neural networks whose output depends not only on the current input values but also on the previous data [32]. In indoor localization, the current location of the target MD is generally correlated to its previous locations as the MD cannot help but move along continuous trajectories. In contrast to the other variations of recurrent neural networks, LSTM can resolve the vanishing gradient problem caused by the gradual reduction of the gradient during the back-propagation process. In addition, LSTM is particularly advantageous in dealing with some practical tasks that exhibit highly correlated features among temporal data, such as machine translation and dialog generation [33]; therefore, we chose the LSTM to fully exploit the correlation among the sequential CSI measurements in each trajectory, which consequently enhances the accuracy of the proposed IPS.

Based on the abovementioned characteristics of 1DCNN and LSTM, the 1DCNN-LSTM architecture was adopted in this paper, as shown in Figure 12. The input of each 1DCNN is a segment of the trajectory CSI in a route composed of multiple RPs from $RP_{x}$ to $RP_{y}$. The entire trajectory CSI with a dimension of $N_{t}\times 56$ in a route, which can be created by concatenating multiple CSI trajectories as described in Section 3.3, is divided into *T* segments, each of which has a dimension of $N_{f}\times 56$ such that $N_{f}\times T=N_{t}$. Note that $N_{t}$ denotes the total number of samples obtained from the entire set of continuously measured trajectory CSI in a given route, $N_{f}$ is the total number of samples allocated in each segment, and *T* is the number of segments that determines the number of 1DCNNs for a given input dataset.

After the trajectory CSI in a route from $RP_{x}$ to $RP_{y}$ is processed in the 1DCNN for each segment, the output of each segment is appended as the input of the corresponding LSTM. In other words, while the 1DCNN is exploited to extract the spatial features from the input trajectory CSI, the LSTM further exploits the resultant spatial information to extract the temporal features. With the information extracted from both spatial and temporal domains, the ambiguity of CSI can be significantly mitigated [34].

The proposed neural network should be trained in such a way that the loss function $L\left(l,\tilde{l}\right)$ that penalizes the Euclidean distance between the output $\tilde{l}$ and target l is minimized. Subsequently, the back-propagation algorithm with the chain rule is applied to calculate the derivative of the loss function $L\left(l,\tilde{l}\right)$ and update the network weights based on the gradient descent. In this paper, we adopt the idea from [35] to train the 1DCNN and LSTM separately; thus, the training of the proposed network is performed in two separate phases. During the first phase, each of the 1DCNNs is trained with the input being the corresponding segment of the given set of trajectory CSI and the loss function, mean square error (MSE), which is defined as:

(5) $$L\left(l-\tilde{l}\right)=\|l-\tilde{l}{\|}_{2}$$

The outputs of the 1DCNNs, {${\bar{c}}_{1}$, ${\bar{c}}_{2}$,…, ${\bar{c}}_{T}$}, are extracted and fed into the corresponding LSTM. During the second phase, the spatial features, {${\bar{c}}_{1}$, ${\bar{c}}_{2},\ldots ,{\bar{c}}_{T}$}, which are the outputs of the 1DCNNs for *T* segments, are used to train the LSTM. The loss function for the second phase is still the MSE for the *T* segments of the MIMO-LSTM model [36] as:

(6) $$L\left(l,\tilde{l}\right)=\frac{\sum_{i=1}^{T}\|l_{i}-{\tilde{l}}_{i}{\|}_{2}}{T}$$

### 5.3. Data Augmentation Using GAN

Although it is desirable to collect as much trajectory CSI as possible for an accurate training of the target neural network, data preparation requires considerable time and effort. To increase the number of training samples without increasing the number of actual observations, we adopted the GAN to efficiently enlarge the training dataset so that the number of observation samples can be significantly increased even with a limited amount of trajectory CSI.

GANs were originally developed [37] to learn the distribution of a given dataset such that a set of synthetic data can be generated with maximum similarity to the real dataset. The GAN used in our paper comprises two distinct neural networks: a generator network and a discriminator network. The former produces a synthetic dataset by learning the distribution of the measured trajectory CSI using a set of noise data of a preset distribution, for example, uniform, Gaussian, etc., such that the distribution of the output matches that of the original trajectory CSI. Assuming both real and synthesized datasets as inputs, the latter indicates whether the two parts of its inputs are discriminable. By iteratively training the generator and discriminator together, the generator’s output, i.e., a set of the synthetic dataset, will become good enough to fool the discriminator. When both the generator and discriminator reach the Nash equilibrium state after enough iterations [38], the synthetic data provided by the generator can no longer be differentiated from the real trajectory CSI with a discriminating probability of approximately 50%.

To generate the synthetic trajectory CSI based on the GAN, we consider the dataset of trajectory CSI obtained from actual observations as follows:

(7) $$R=\left(\begin{array}{ccc} r_{11}\;r_{12}\;r_{21}\;r_{22} & \cdots & r_{1M}\;r_{2M}\\ \vdots & \ddots & \vdots \\ r_{K1}\;r_{K2} & \cdots & r_{KM} \end{array}\right),$$

where *M* is the number of time steps per trajectory and *K* is the number of data measurements per trajectory, with $r_{ij}$ being the amplitude obtained from the ith measurement at the jth time step. Let vector $x\in {\mathbb{R}}^{1\times M}$ denote the synthesized data provided from the generator; it can be written as $x=\left[x_{1},x_{2,\;\ldots ,\;}x_{M}\;\right]$. Meanwhile, the prior noise latent variable $z\in {\mathbb{R}}^{1\times L}$, the input of the generator can be written as $z=\left[z_{1},z_{2,\;\ldots ,\;}z_{L}\;\right]$, where *L* is the dimension of the latent space.

**Figure 13:**
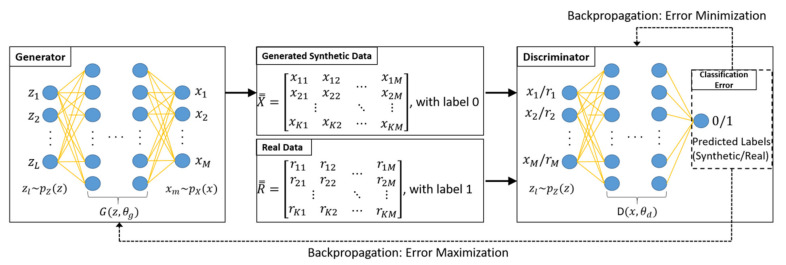
Proposed GAN structure for generating synthetic data that can fool the discriminator.

Figure 13 illustrates the proposed GAN structure. The process for producing synthetic data is based on the following loss function:

(8) $$\begin{array}{c} Min\\ G \end{array}\;\begin{array}{c} Max\\ D \end{array}L\left(D,\;G\right)=E\left[logD\left(x\right)\right]+E[\log(1-D\left(G\left(z\right)\right))],$$

where $E\left[\cdot \right]$ denotes the expectation of a random process with the distribution function of data x and noise *z* being p(*x*) and p(*z*)*,* respectively. The loss function comprises two terms, including the generator and discriminator function denoted by G and D, respectively. Both G and D are differentiable functions represented by a multilayer perceptron (MLP). During the training period, the generator learns how to match the distribution of latent noise z, which has been arbitrarily set to $P_{Z}\left(z\right)$, with that of the real data, $P_{R}\left(r\right)$, using the $G\left(z,\;\theta_{g}\right)$ structure, where $\theta_{g}$ denotes the parameters of the MLP in the generator. In contrast to the generator, the discriminator learns during its training period how to classify the real and synthetic data using the $D\left(x,\;\theta_{d}\right)$ structure, where $\theta_{d}$ denotes the parameters of the MLP in the discriminator. The discriminator produces either 0 or 1 as its output, indicating synthetic or real data, respectively.

The Adam optimizer [39] was used to update the generator and discriminator parameters adopted in the proposed neural network. Convergence occurs when $D\left(x,\;\theta_{d}\right)$ becomes 1/2, meaning that the discriminator cannot distinguish the real and synthetic data from each other. After the convergence of both the generator and discriminator, the generator is ready to produce a synthetic dataset with its distribution function being the same as that of the original measured trajectory CSI.

## 6. Experimental Results

The proposed deep learning models of 1DCNN-LSTM and GAN were implemented with TensorFlow 2.0 on an NVidia RTX 2080Ti GPU using the Ubuntu 20.04 operating system. The dataset for training and testing the neural networks was obtained from the proposed IPS—the hardware implementation is detailed in Section 4.

### 6.1. Dataset of Trajectory CSI for Experiments

As mentioned in Section 3, our dataset was obtained from 20 trajectories in five routes, with 21 RPs in total. Each route comprises four trajectories, which brings about the 20 trajectories in total for our experiments. For each trajectory, the trajectory CSI was measured 100 times using the proposed data collection method described in Section 3. The dimension of the data samples of each trajectory CSI is set to be 2000 × 56 because we have 2000 CSI samples from each of the continuously measured CSI trajectories, with each sample comprising 56 subcarriers, as shown in Figure 7. Therefore, there are 20 data groups corresponding to 20 trajectories in the dataset. Each data group is labeled with the corresponding trajectories (0~19).

Among the 100 measurements for each trajectory, one out of every five trajectory CSI measurements were selected to build the test dataset, whereas the remaining measurements were the training dataset. Consequently, our dataset comprises 80 measurements of the trajectory CSI for the training dataset and 20 for the test dataset. The 100 measurements for each trajectory must be shuffled well enough such that the training data can be allocated as uniformly as possible over the entire observation period. Consequently, the total number of samples in the training data set is 8,960,000 per trajectory (=80 measurements of 2000 samples for each of 56 subcarriers) while that of the test dataset is 2,240,000 per trajectory (=20 measurements of 2000 samples for each of 56 subcarriers). By using the abovementioned training and test datasets, the proposed deep learning networks are trained and tested to construct the optimal IPS.

### 6.2. Impact of Convolutional Filter Dimension of 1DCNN

To achieve the best-optimized model for the 1DCNN, we conducted experiments to evaluate the impact of the convolutional filter dimension on the 1DCNN performance based on the number of convolutional layers and filters.

Table 1 shows a comparison of 1DCNN performances based on different numbers of filters and different numbers of layers—one can observe that the accuracy is generally enhanced as the number of layers increases. This is because the learning capability is improved as the number of convolutional layers increases. In addition, as the number of filters increases from 16 to 64, the mean distance error decreases from 1.94 to 1.74 m; however, as the number of filters increases to 128, the mean distance error of the IPS worsens to 2.24 m. This indicates that an excessively large number of filters may result in worse IPS performance because it makes the training of the neural network more difficult.

We also observed the performance of the IPS as a function of the number of convolutional layers with various numbers of convolutional filters employed at each layer. Through extensive experiments, we found that the 256-128-64-32 configuration provides the lowest mean distance error, i.e., 1.34 m, among all the observed configurations. However, the training time for this configuration, i.e., 268 s, was much longer than that for most other configurations because of its four-layered structure with numerous convolutional filters. In the case of both the mean distance error and training time, we found that the configuration of 64-32 provides reasonably good performance with a mean distance error of 1.35 m, which is only 0.75% higher than that of the configuration of 256-128-64-32, and the training time is 134 s, which is approximately 50% faster than that of the configuration of 256-128-64-32.

From the above experiments, we observed that a double-layered model with 64 filters in the first layer and 32 filters in the second layer provided the best performance in terms of both the mean distance error and training time in the indoor experimental environment described in Section 4. Although a 1DCNN with more than two layers may provide similar or even slightly better mean distance error performance, it inherently leads to a long training time because of the challenge of training the given neural networks. Consequently, a 1DCNN with fewer layers and a small number of filters in each layer might be preferred in terms of the training speed. Table 2 lists the detailed parameters of the proposed 1DCNN structure.

### 6.3. Impact of the Number of Segments T

The number of segments *T* as described in Section 5.2 increases when the concatenated trajectory CSI generated by concatenating a set of trajectory CSI in a predetermined route is divided into more segments. As the number of segments *T* increases, more 1DCNNs are utilized to extract features from the corresponding segment. To find the optimal *T* for the 1DCNN-LSTM architecture, we conducted extensive experimental tests on different values of *T*. The detailed parameters for each LSTM of the proposed IPS are listed in Table 3.

The performance of the IPS based on *T* is summarized in Table 4. It can be observed that the localization mean distance error is enhanced from 1.59 to 1.18 m as T increases from 1 to 4. In our experiments, it was found that the best performance was obtained when T was set to 5, yielding a mean distance error and standard deviation of 0.96 and 0.63 m, respectively. However, as T increases beyond 5, the performance of the IPS degrades, which indicates that numerous segments do not necessarily provide better performance, owing to the accumulated errors at each segment. Therefore, segments T should be sufficiently large to obtain sufficient information from the previous segments. If the value of T is too large, then it is more likely that invalid information can be generated from at least one LSTM, which will result in inaccurate predictions for all the following LSTMs.

### 6.4. Impact of the Number of Units in LSTM

For each LSTM, the number of units, which is related to the capacity of the LSTM for learning the input data, must be set up with an appropriate value in such a way that it causes neither over-fitting with an excessively large value nor under-fitting with an excessively small value. Thus, the tuning of this hyperparameter is generally determined empirically through experiments.

**Figure 14:**
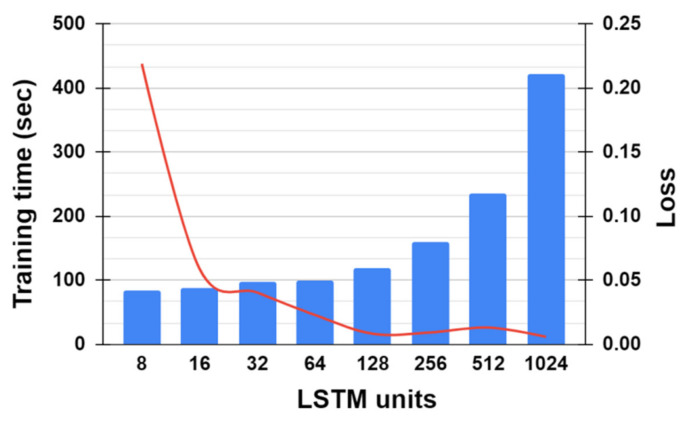
Effect of the number of LSTM units on the training time (denoted by the blue bar) and loss (denoted by the red curve) of 1DCNN-LSTM.

Figure 14 shows how the number of units in each LSTM results in the training time and loss. The loss of the model during the training period decreases as the number of units employed in LSTM increases. However, when the number of units increases beyond 128, the training time rapidly increases due to numerous parameters being trained, whereas the loss is not decreased conspicuously anymore. For instance, when the number of units is 1024, the training time almost doubles compared to when the number of units is 128, where the loss is almost constant. The trade-off between the training time and loss has led to the conclusion that 128 is the optimal value for the number of units in each LSTM for our model based on our extensive experiments.

### 6.5. Impact of the Batch Size

The number of samples in the training dataset used to estimate the gradient of the loss before the model weights are updated is denoted as the batch size [40]. It is an important hyperparameter that should be well tuned to optimize the system’s dynamics, such as the training speed and stability of the learning process.

**Figure 15:**
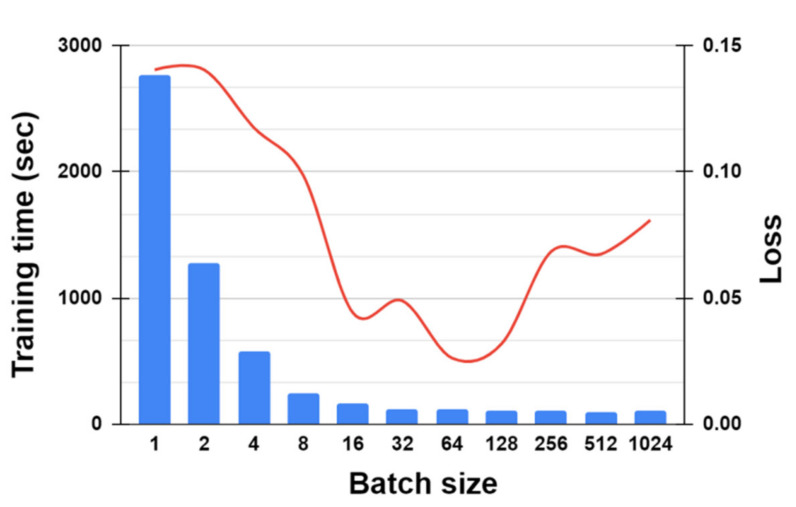
Effect of batch size on the training time (denoted by the blue bar) and loss (denoted by the red curve) of 1DCNN-LSTM.

Figure 15 shows how the batch size impacts the training time and loss. As the batch size increases from 1 to 1024, the time required for training the 1DCNN-LSTM is decreased exponentially. As the batch size increases beyond 32, the training time converges nearly to 100 s. Conversely, the loss of the model decreases from 0.1405 to 0.0261 as the batch size increases from 1 to 64, owing to the increase of the speed for updating the weights of the model; however, as the batch size increases beyond 64 (up to 1024), the loss increases, owing to the poor generalization of the mapping between the input and output of the model. The results of the extensive experiments show that the optimal value for the batch size of our model was 64.

### 6.6. Impact of the Number of Trajectories

The objective of this subsection is to verify that the route prediction accuracy is improved as we increase the amount of input data of the proposed neural network by concatenating several consecutive trajectory CSI of a given route. With the five routes predetermined, as shown in Figure 9, the proposed IPS predicts which route among the five predetermined routes the test trajectory/trajectories belong to.

**Figure 16:**
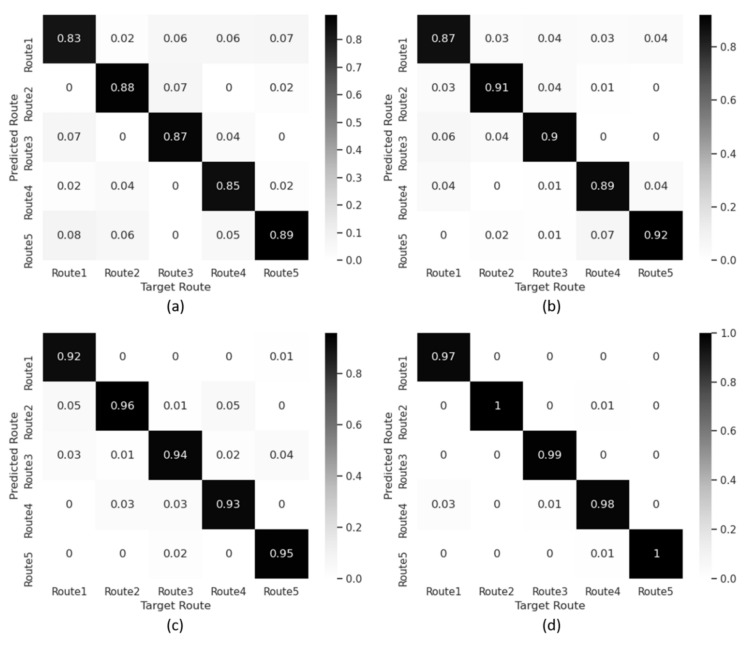
Confusion matrix of route prediction accuracy according to different numbers of trajectories. (**a**) Route prediction accuracy with one trajectory. (**b**) Route prediction accuracy with two trajectories. (**c**) Route prediction accuracy with three trajectories. (**d**) Route prediction accuracy with four trajectories.

Figure 16 illustrates confusion matrices representing the route prediction accuracy according to the number of trajectories, where Figure 16a–d represent the results obtained by using the proposed 1DCNN-LSTM, with the number of input trajectories being one, two, three, and four, respectively. It can be easily observed that the probability for the correct route prediction increases as the number of trajectories employed as an input of the proposed IPS increases. It is also noteworthy that all the trajectory CSI that has been already used should not be discarded from the input dataset of the neural network in order to be reused together with the trajectory CSI to be collected later.

### 6.7. Performance Analysis on GAN

The objective of this subsection is to present the performance of the proposed IPS, which employs the synthetic data provided by the GAN model discussed in Section 5. More specifically, the performance is analyzed as a function of the portion of synthetic data added to the training dataset described in Section 6.

To observe how the synthetic data provided by the GAN model enhance the performance of the proposed IPS, we performed two experiments as follows. In the first experiment, 20% (i.e., 16 measurements) of the entire training data was randomly selected and presented to the GAN model to generate a set of synthetic data, which was added to the dataset of the 16 real measurements to increase the total quantity of the training dataset. Subsequently, the performance of the proposed IPS was analyzed as a function of the number of synthetic data added to the 16 real measurements. In the second experiment, we performed the same experiments as the first, except that all the training data (i.e., 80 measurements) were used along with the synthetic data as the training dataset. Table 5 presents the performance of the proposed IPS in terms of test accuracy and log-likelihood loss based on the number of synthetic data employed together with the actual measurements. The accuracy shown in Table 5 is defined as ($\frac{N_{true}}{N_{total}}\times 100\%$), where $N_{true\;}$ is the number of true predictions for the test data and $N_{total}$ is the total number of trajectories CSI, which is 20 (the total number of trajectories) × 20 (the total number of test samples per trajectory) in our experiments. As Table 5 shows, the accuracy of the IPS is only 71.2% when the 16 real training measurements were used with no synthetic data for training the neural network. When all 80 real training measurements were used without the synthetic data for training the neural network, the accuracy was 93.3%, which indicates that the IPS performed better when more training data were provided. However, as we added 100 synthetic data generated from the 16 samples using the GAN, the accuracy of the IPS was significantly improved from 71.2% to 93.8%, which is nearly equivalent to the case of the IPS trained with the 80 real measurements. As the amount of synthetic data added to the training dataset increases, the accuracy of the IPS is enhanced and then saturated after adding approximately 300 synthetic data to 20% of the real data, i.e., 16 measurements.

Table 5 shows the accuracy of the IPS when the dataset consists of 20% real data and 80% synthetic data generated using GAN. We also conducted an experiment on the relationship between the classification accuracy and the percentage of real data used for generating synthetic data using GAN; this experiment shows how many real measurements are actually required to generate synthetic training data that can efficiently enhance the IPS performance.

**Figure 17:**
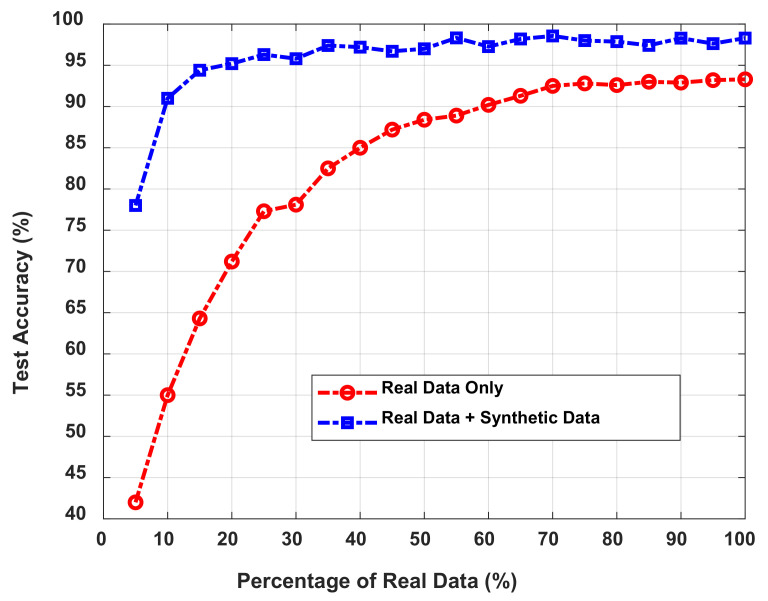
Comparison of classification accuracy between real data only (**blue line**) and real data combined with synthetic data (**red line**).

Figure 17 illustrates a comparison of classification accuracy between using real data only and using real data combined with synthetic data. In our experiments, as shown in Figure 17, the test accuracy increases as the number of real measurements used for generating the synthetic data increases from 4 to 80, which is 5–100% of the 80 real training measurements. The accuracy is only 42% when 5% of the real measurements are used to train the GAN. By adding synthetic data generated with 5% of the real measurements, the accuracy is enhanced to 78%. As the percentage of real data used to generate synthetic data increased from 5% to 25%, the accuracy of the IPS is enhanced from 78% to 96.3%, which is significantly higher than that of the IPS trained only with the real data. It can also be observed that more than 35% of real data (28 samples) being used by the GAN does not conspicuously enhance the accuracy, meaning the GAN employed in the proposed IPS can provide enough valid synthetic data with only 35% of the real trajectory CSI.

### 6.8. Performance Comparison with State-of-the-Art Methods

State-of-the-art neural networks, i.e., ConFi [21], DeepFi [16], and Horus [41], were used to compare with the proposed neural networks, i.e., 1DCNN-LSTM aided by the GAN, to evaluate the performance of the proposed IPS. The optimized configurations obtained from our extensive experiments were used for 1DCNN-LSTM. To be more specific, there were two convolutional layers with 64 filters in the first layer and 32 filters in the second layer for each 1DCNN, the number of units in each LSTM was 128, and there were five segments.

**Figure 18:**
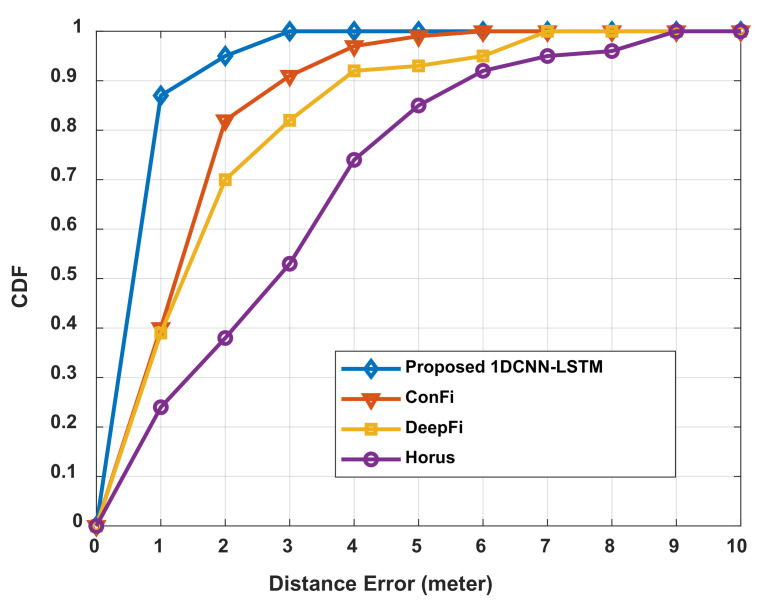
CDF of the localization error of the proposed 1DCNN-LSTM.

Table 6 shows the numerical results obtained by the proposed method and other state-of-the-art methods. The best performance is observed from the proposed 1DCNN-LSTM model with the mean distance error being 0.74 m, outperforming the ConFi, DeepFi, and Horus methods by 46.0%, 47.9%, and 61.9%, respectively. Figure 18 shows the cumulative distribution function for all four methods—we see that the proposed 1DCNN-LSTM outperforms ConFi, DeepFi, and Horus with a distance error probability of 87% within 1 m, 95% within 2 m, and 100% within 3 m.

### 6.9. Performance Comparison with Different Spacing between RPs in Two Different Signal Environments

**Figure 19:**
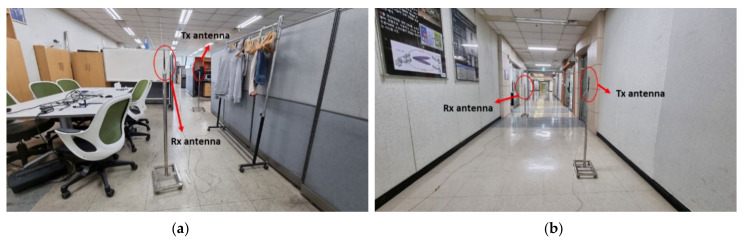
Experimental environments (**a**) Narrow laboratory with various furniture. (**b**) Relatively wide corridor.

The objective of this subsection is to present the performance of the proposed IPS with a few different selections for the spacing between RPs in two different signal environments. The two different indoor signal environments are (1) narrow laboratory with various furniture, and (2) relatively wide corridor, of which the photos are shown in Figure 19a,b, respectively.

Table 7 shows the numerical results obtained from experimental tests using the proposed method in the abovementioned two different indoor signal environments with four different values for the spacing, 60 cm, 80 cm, 100 cm, and 120 cm. First of all, it has been observed that the performances in the two different signal environments are quite different from each other. The performance obtained in the wide corridor is much better than that of the narrow laboratory. The main reason is that the Wi-Fi signal in the narrow laboratory suffers more severely than that of the wide corridor from adverse multipath effects. The lowest mean error is 0.73 m with the spacing being 60 cm in the laboratory signal environment, whereas the lowest mean error is 0.43 m with the spacing being 100 cm in the corridor signal environment. Second of all, it has been observed that, for both laboratory and corridor signal environments, the value for the inter-RP spacing turns out to be not a major factor affecting the performance of the proposed IPS, which should be taken as granted because our method is based on the trajectory CSI continuously measured in between adjacent RPs instead of the single-point CSI measured at each RP. It means that the proposed IPS exploits all the CSI values between RPs, which in other words indicates that the effective value for the spacing in the proposed IPS is nearly 0.

## 7. Conclusions

In this paper, we proposed a novel IPS using the trajectory CSI continuously collected along each trajectory. Compared to the traditional method of collecting CSI measurements at predetermined fixed locations, our proposed trajectory CSI collection method can collect continuous CSI existing in the trajectories, and utilize it to achieve high accuracy and reliability in localization. The proposed IPS was implemented using multicore DSP and USRP as a modem and RF transceiver, respectively, to emulate Wi-Fi APs and MDs supporting the IEEE 802.11ac protocol, and using a GPU as a training device for the proposed system. For the deep learning solution, we adopted a 1DCNN-LSTM deep learning architecture, which can extract both spatial and temporal information from the given trajectory CSI to predict the MD’s current location with the help of the information provided from previous data. Additionally, to tackle the challenge of obtaining sufficient trajectory CSI, the GAN was employed in this paper to generate synthetic data with only a small amount of real trajectory CSI measurements. Through extensive onsite experiments in an indoor environment, the results consistently demonstrate that our 1DCNN-LSTM deep learning structure trained with the training dataset comprising both real and synthetic data generated by the GAN achieves an average localization error of 0.74 m, which outperforms the state-of-the-art algorithms ConFi by 46.0%, DeepFi by 47.9%, and Horus by 61.9%.

## Figures and Tables

**Table 1:** Comparison of 1DCNN performances for different configuration of convolutional filters.

# of Convolutional Filters	Mean Error (m)	Std. Dev. (m)	Training Time (s)
16 (Single layer)	1.94	1.24	113
32 (Single layer)	1.85	1.43	110
64 (Single layer)	1.74	1.14	121
128 (Single layer)	2.24	1.18	143
256 (Single layer)	2.91	1.86	172
32-16 (Double layer)	1.83	1.17	118
64-32 (Double layer)	1.35	0.90	134
64-32-16 (Triple layer)	1.49	0.95	137
128-64 (Double layer)	3.33	2.13	163
128-64-32 (Triple layer)	1.36	0.71	174
128-64-32-16 (Quad. layer)	1.39	0.82	178
256-128 (Double layer)	2.47	1.58	235
256-128-64 (Triple layer)	1.37	0.88	259
256-128-64-32 (Quad. layer)	1.34	0.86	268
256-128-64-32-16 (Quint. layer)	1.39	0.89	274

**Table 2:** 1DCNN layer parameters.

Configuration	Value
Training Data Size	2000 [samples] × 56 [subcarriers] × 80 [# of measurements]
Test Data Size	2000 × 56 × 20
Conv Layer 1	Input (2000 × 56 × 1), 3 × 3 kernels, 64 filters
Conv Layer 2	Input (2000 × 56 × 64), 3 × 3 kernels, 32 filters
Fully Connected Layer	16 neurons
Training Output	Location $\tilde{l}$
Loss Function	MSE
Optimizer	Adam
Learning rate	0.001

**Table 3:** LSTM parameters.

Configuration	Value
Loss function	MSE
Fully connected Layer	16 neurons
Dropout	0.2
Optimizer	Adam
Learning rate	0.001

**Table 4:** LSTM parameters performance comparison according to the number of segments.

# CSI Segments (T)	Mean Error (m)	Std. Dev. (m)
1	1.59	1.02
2	1.42	0.91
3	1.35	0.86
4	1.18	0.76
5	0.96	0.63
6	1.10	0.71
7	1.68	1.08
8	2.18	1.40
9	2.76	1.77
10	3.19	2.04

**Table 5:** Test accuracy and log-likelihood loss of adding synthetic data samples to 20% and 100% of real data.

	Real Data	20% (16 Samples)		100% (80 Samples)	
Synthetic Data		Accuracy	Log Loss	Accuracy	Log Loss
0		71.2%	1.43	93.3%	0.13
100		93.8%	0.13	96.7%	0.08
200		95.0%	0.11	97.4%	0.07
300		95.2%	0.09	98.3%	0.06
400		95.1%	0.09	98.2%	0.06

**Table 6:** Performance comparison between proposed method and existing methods for indoor environments.

	Mean Error (m)	Std.Dev. (m)	50th Pctl. (m)	90th Pctl. (m)
1DCNN-LSTM	0.74	0.43	0.58	1.43
ConFi	1.37	0.97	1.26	2.82
DeepFi	1.42	1.02	1.43	3.79
Horus	1.94	1.53	2.78	5.76

**Table 7:** IPS performance comparison according to the spacing between adjacent RPs and experimental environments.

	Experimental Environment	Laboratory		Corridor	
Spacing (cm)		Mean Error (m)	Std. Dev. (m)	Mean Error (m)	Std. Dev. (m)
60		0.73	0.42	0.45	0.26
80		0.74	0.43	0.44	0.25
100		0.76	0.46	0.43	0.23
120		0.74	0.43	0.47	0.27

## References

[1] Batalla J.M., Mavromoustakis C.X., Mastorakis G., Xiong N.N., Wozniak J. (2020). Adaptive Positioning Systems Based on Multiple Wireless Interfaces for Industrial IoT in Harsh Manufacturing Environments. IEEE J. Sel. Areas Commun..

[2] Basiri A., Lohan E.S., Moore T., Winstanley A., Peltola P., Hill C., Amirian P., e Silva P.F. (2017). Indoor location based services challenges, requirements and usability of current solutions. Comput. Sci. Rev..

[3] Li H., He X., Chen X., Fang Y., Fang Q. (2019). Wi-Motion: A Robust Human Activity Recognition Using WiFi Signals. IEEE Access.

[4] Zheng L., Hu B., Chen H. (2018). A High Accuracy Time-Reversal Based WiFi Indoor Localization Approach with a Single Antenna. Sensors.

[5] Nessa A., Adhikari B., Hussain F., Fernando X.N. (2020). A Survey of Machine Learning for Indoor Positioning. IEEE Access.

[6] Khalajmehrabadi A., Gatsis N., Akopian D. (2017). Modern WLAN fingerprinting indoor positioning methods and deployment challenges. IEEE Commun. Surv. Tutor..

[7] Duan Y., Lam K.-Y., Lee V.C., Nie W., Liu K., Li H., Xue C.J. (2019). Data Rate Fingerprinting: A WLAN-Based Indoor Positioning Technique for Passive Localization. IEEE Sens. J..

[8] Al-Qaness M.A.A., Li F. (2016). WiGeR: WiFi-based gesture recognition system. Int. J. Geo-Inf..

[9] Yousefi S., Narui H., Dayal S., Ermon S., Valaee S. (2017). A Survey on Behavior Recognition Using WiFi Channel State Information. IEEE Commun. Mag..

[10] Ali M.U., Hur S., Park Y. (2017). LOCALI: Calibration-Free Systematic Localization Approach for Indoor Positioning. Sensors.

[11] Yang Z., Zhou Z., Liu Y. (2013). From RSSI to CSI: Indoor localization via channel response. ACM Comput. Surv..

[12] Wu K., Xiao J., Yi Y., Chen D., Luo X., Ni L.M.-S. (2012). CSI-Based Indoor Localization. IEEE Trans. Parallel Distrib. Syst..

[13] Zhang L., Ding E., Hu Y., Liu Y. (2019). A novel CSI-based fingerprinting for localization with a single AP. EURASIP J. Wirel. Commun. Netw..

[14] Dang X., Tang X., Hao Z., Liu Y. (2019). A Device-Free Indoor Localization Method Using CSI with Wi-Fi Signals. Sensors.

[15] Yin Y., Song C., Li M., Niu Q. (2019). A CSI-Based Indoor Fingerprinting Localization with Model Integration Approach. Sensors.

[16] Wang X., Gao L., Mao S., Pandey S. (2016). CSI-based Fingerprinting for Indoor Localization: A Deep Learning Approach. IEEE Trans. Veh. Technol..

[17] Schmidt E., Inupakutika D., Mundlamuri R., Akopian D. (2019). SDR-Fi: Deep-Learning-Based Indoor Positioning via Software-Defined Radio. IEEE Access.

[18] Shi S., Sigg S., Chen L., Ji Y. (2018). Accurate Location Tracking from CSI-Based Passive Device-Free Probabilistic Fingerprinting. IEEE Trans. Veh. Technol..

[19] Hsieh C.-H., Chen J.-Y., Nien B.-H. (2019). Deep Learning-Based Indoor Localization Using Received Signal Strength and Channel State Information. IEEE Access.

[20] Wang F., Feng J., Zhao Y., Zhang X., Zhang S., Han J. (2019). Joint Activity Recognition and Indoor Localization with WiFi Fingerprints. IEEE Access.

[21] Chen H., Zhang Y., Li W., Tao X., Zhang P. (2017). ConFi: Convolutional neural networks based indoor Wi-Fi localization using channel state information. IEEE Access.

[22] Gu Z., Chen Z., Zhang Y., Zhu Y., Lu M., Chen A. (2016). Reducing fingerprint collection for indoor localization. Comput. Commun..

[23] Pulkkinen T., Roos T., Myllymaki P. (2011). Semi-supervised learning for wlan positioning. Proceedings of the 21th International Conference on Artificial Neural Networks—Volume Part I, ser. ICANN’11.

[24] Liu S., Luo H., Zou S. A Low-Cost and Accurate Indoor Localization Algorithm Using Label Propagation Based Semi-supervised Learning. Proceedings of the 2009 Fifth International Conference on Mobile Ad-hoc and Sensor Networks.

[25] Sung C.K., De Hoog F., Chen Z., Cheng P., Popescu D.C. (2017). Interference Mitigation Based on Bayesian Compressive Sensing for Wireless Localization Systems in Unlicensed Band. IEEE Trans. Veh. Technol..

[26] Cohen I., Huang Y., Chen J., Benesty J. (2009). Pearson correlation coefficient. Noise Reduction in Speech Processing.

[27] IEEE (2013). IEEE Standard for Information Technology—Telecommunications and Information Exchange between Systems—Local and Metropolitan Area Networks—Specific Requirements. Part 11: Wireless LAN Medium Access Control (MAC) and Physical Layer (PHY) Specific Requirements.

[28] Texas Instruments TMS320C6670 Multicore Fixed and Floating-Point System-on-Chip Data Manual. http://www.ti.com/lit/ds/symlink/tms320c6670.pdf.

[29] Ettus Resarch USRP X310 Data Sheet. https://www.ettus.com/wp-content/uploads/2019/01/X300_X310_Spec_Sheet.pdf.

[30] Shao W., Luof H., Zhao F., Wang C., Crivello A., Tunio M.Z. DePos: Accurate orientation-Free Indoor Positioning with Deep Convolutional Neural Networks. Proceedings of the 2018 Ubiquitous Positioning, Indoor Navigation and Location-Based Services (UPINLBS).

[31] Hochreiter S., Schmidhuber J. (1997). Long short-term memory. Neural. Comput..

[32] Graves A., Mohamed A.-R., Hinton G. Speech recognition with deep recurrent neural networks. Proceedings of the 2013 IEEE International Conference on Acoustics, Speech and Signal Processing.

[33] Sagheer A., Kotb M. (2018). Time series forecasting of petroleum production using deep LSTM recurrent networks. Neurocomputing.

[34] Hoang M.T., Yuen B., Ren K., Dong X., Lu T., Westendorp R., Reddy K. (2020). A CNN-LSTM Quantifier for Single Access Point CSI Indoor Localization. arXiv.

[35] Wu Z., Wang X., Jiang Y.-G., Ye H., Xue X. (2015). Modeling Spatial-Temporal Clues in a Hybrid Deep Learning Framework for Video Classification. Proceedings of the 23rd ACM International Conference on MULTIMEDIA.

[36] Hoang M.T., Yuen B., Dong X., Lu T., Westendorp R., Reddy K. (2019). Recurrent Neural Networks for Accurate RSSI Indoor Localization. IEEE Internet Things J..

[37] Goodfellow I., Pouget-Abadie J., Mirza M., Xu B., Warde-Farley D., Ozair S., Courville A., Bengio Y. Generative adversarial nets. Proceedings of the Advances in Neural Information Processing Systems 2014.

[38] Cao Y.-J., Jia L.-L., Chen Y.-X., Lin N., Yang C., Zhang B., Liu Z., Li X.-X., Dai H.-H. (2018). Recent Advances of Generative Adversarial Networks in Computer Vision. IEEE Access.

[39] Kingma D., Ba J. (2014). Adam: A method for Stochastic Optimization. arXiv.

[40] Smith S.L., Kindermans P.-J., Le Q.V. (2017). Don’t decay the learning rate, increase the batch size. arXiv.

[41] Youssef M., Agrawala A. (2005). The Horus WLAN location determi- nation system. Proceedings of the 3rd International Conference on Mobile Systems, Applications, and Services.

